# Genome-Wide Identification and Analysis of *bZIP* Gene Family and Resistance of *TaABI5* (*TabZIP96*) under Freezing Stress in Wheat (*Triticum aestivum*)

**DOI:** 10.3390/ijms23042351

**Published:** 2022-02-21

**Authors:** Yi Liang, Jingqiu Xia, Yunshuang Jiang, Yuzhuo Bao, Huichan Chen, Duojia Wang, Da Zhang, Jing Yu, Jing Cang

**Affiliations:** College of Life Science, Northeast Agricultural University, Harbin 150030, China; neaulyi@163.com (Y.L.); xiajingqiu175@gmail.com (J.X.); jiangyunshuang@gmail.com (Y.J.); baoyuzhuo1230@163.com (Y.B.); chenhuichan1996@gmail.com (H.C.); wangduojia1234@gmail.com (D.W.); zhangda@neau.edu.cn (D.Z.); yujing1678@gmail.com (J.Y.)

**Keywords:** *Triticum aestivum*, *bZIP* gene, *TaABI5*, freezing stress, functional verification

## Abstract

The basic leucine zipper (*bZIP*) regulates plant growth and responds to stress as a key transcription factor of the Abscisic acid (ABA) signaling pathway. In this study, *TabZIP* genes were identified in wheat and the gene structure, physicochemical properties, cis-acting elements, and gene collinearity were analyzed. RNA-Seq and qRT-PCR analysis showed that ABA and abiotic stress induced most *TabZIP* genes expression. The ectopic expression of *TaABI5* up-regulated the expression of several cold-responsive genes in *Arabidopsis*. Physiological indexes of seedlings of different lines under freezing stress showed that *TaABI5* enhanced the freezing tolerance of plants. Subcellular localization showed that *TaABI5* is localized in the nucleus. Furthermore, TaABI5 physically interacted with cold-resistant transcription factor TaICE1 in yeast two-hybrid system. In conclusion, this study identified and analyzed members of the *TabZIP* gene family in wheat. It proved for the first time that the gene *TaABI5* affected the cold tolerance of transgenic plants and was convenient for us to understand the cold resistance molecular mechanism of *TaABI5*. These results will provide a new inspiration for further study on improving plant abiotic stress resistance.

## 1. Introduction

Wheat is one of the most important crops and is an important plant protein resource around the world. Low temperature stresses will seriously affect wheat’s growth state and then lead to yield reduction. Chilling stress and freezing stress can damage cell membranes and increase ROS in the body, thus affecting the growth and physiological state of plants, especially the yield and quality of wheat and other field crops [1]. Under the stress condition, plants can produce stress response to adapt to the adverse factors through a variety of pathways, so as to reduce the negative influence of the environment on themselves [2]. Plant transcription factors (TFs) play crucial roles in the regulatory and biological processes under various environmental stresses [3]. TFs are sequence-specific binding proteins that bind to the promoter regions of specific target genes to regulate their transcription [4,5]. It controls cell processes such as signal transduction, cell morphogenesis and resistance to environmental stress in plants [6,7].

The basic leucine zipper (*bZIP*) transcription factors family member is one of the most diverse and largest transcription factors in eukaryotes [8,9]. Leucine zipper is a dimerization motif, consisting of a helical structure composed of seven nucleotide repeats of leucine or other large hydrophobic amino acids. These helical structures ensure the stability and specificity of the dimerization reaction [10]. The domain of bZIP is 60 to 80 amino acids in length and contains two regions with diverse functions [3]. And DNA binding base region is highly conserved, with an invariant N-x7-R/K motif. Plant *bZIP* protein preferentially binds to the DNA sequence of ACGT core, such as A-box (TACGTA), C-box (GACGTC), and G-box (CACGTG). The binding specificity is regulated by flanking nucleotides [11].

To date, the *bZIP* gene family in many plants has been extensively studied. The *bZIP* genes are involved in important regulatory processes of plant growth and development, such as regulating root development [12], promoting anthocyanin accumulatio [13], and integrating transient abscisic acid and glucose signals [14]. Similarly, *bZIP* genes also participate in regulating the physiological responses of plants under stress. *bZIP* genes can affect salt and drought tolerance of many species, such as tomato [15], rice [16], soybean [17], and panax ginseng [18]. The cold signal of pear is regulated by *PbbZIP* [19]. *mrna30280* and *mrna11827*, as *FvbZIP* genes, are involved in the heat stress response pathway [20]. A variety of bZIP proteins are also involved in regulating different hormones. MabZIP74 can interact with MaMAPK11-3 and regulate the expression of ethylene synthesis gene *MAaco1/4* in bananas [21] and *OsbZIP81* positively regulates JA levels by targeting JA regulatory genes [22]. The bZIP transcription factor *BZI-1* of tobacco is involved in auxin-mediated growth [23]. *VvbZIP60* contributes importantly to SA signaling against by up-regulating *PR1* in grape [24].

The *bZIP* gene in wheat also has a variety of biological functions. Overexpression of *TabZIP28* enhanced heat tolerance of transgenic *Arabidopsis thaliana*. [25]. As a positive regulator of wheat stripe rust resistance, *TabZIP74* is involved in root development through mRNA splicing [26]. *TabZIP3* is involved in the salt stress response of wheat [27]. As a *bZIP* transcription factor, *TaABP1* had better drought resistance and was strongly expressed under ABA treatment [28]. *TabZIP15* can improve salt tolerance of wheat [29]. TaFDL2 interacts with TabZIP8 protein to jointly regulate ABA signaling in response to drought stress [30]. The osmotic stress tolerance of wheat was closely related to the expression level and trend of *TaDREB1-D* [31]. *TabZIP14-B* is involved in salt tolerance and frost resistance in plants [32]. These results suggest that bZIP transcription factors are involved in various hormone regulation in different species, and the *TabZIP* gene may affect plant growth and stress tolerance through hormonal signaling pathways.

Representatively, Abscisic acid (ABA) has often been reckoned as the universal stress phytohormone due to its significant influence on plant growth regulation during biotic and abiotic stress responses [33]. The bZIP transcription factor in plants is the largest of the known ABA-induced DNA-binding protein family and can effectively trigger downstream genes during abiotic stress [8]. ABA senses stress signals and forms a complex with PYL/PYL/RCAR receptor and PP2C phosphatase, preventing PP2C phosphatase from dephosphorylating SnRK2 kinase, resulting in phosphorylation and activation of bZIP transcription factor by SnRK2 kinase, such as ABA-insensitive 5 (ABI5) and ABRE binding factor/ABRE binding protein (ABFs/AREBs) [34]. Thereinto, ABI5 (ABA-INSENSITIVE 5) was named after the fifth ABA-non-responsive mutant identified by Finkelstein et al. [35]. As a key transcription factor in the ABA signaling pathway, ABI5 is also involved in plant growth [36] and metabolism [37]. Simultaneously, it is also involved in the regulation of various stress responses. For instance, *AtABI5* can improve transgenic cotton resistance to drought stress by regulating ROS scavenging [38]. Overexpression of AtABI5 increased the accumulation of DGAT1 and TAG proteins, and regulated the growth of *Arabidopsis thaliana* seedlings under stress [39]. *ABI5* can improve the drought resistance of rice [40] and barley [41]. The salt tolerance of Siberian wildrye [42] and maize [43] was also regulated by *ABI5*. Studies in several species have shown that the response and regulation of *ABI5* are mainly reflected in osmotic stress such as drought and salt, but there are few reports on the cold resistance mechanism of wheat.

In this study, 227 members of the *TabZIP* gene family were identified and divided into nine groups. *TaABI5*, as a representative of group A, showed excellent performance under various stresses. Therefore, we cloned the *TaABI5* gene, studied its role in cold resistance through ectopic expression in *Arabidopsis* and further explored the low-temperature resistance mechanism of TaABI5 by interacting with a cold-resistance transcription factor TaICE1 [44] protein. The resistance of transgenic lines and wild-type plants to abiotic stress was evaluated by several physiological parameters. This study will provide a theoretical reference for the study of *bZIP* genes in plant abiotic resistance.

## 2. Results

### 2.1. Identification of bZIP Gene Family in Wheat

After removing erroneous and redundant sequences, 227 *TabZIP* genes were identified for subsequent study. Nomenclature of these genes was given based on the chromosome position, and all genes were renamed from *TabZIP1* to *TabZIP227*. The basic information of 227 *TabZIP* genes was analyzed in this study, including the protein sequence lengths, relative molecular weight (MW), and isoelectric point (pI). As shown in Table S1, the amino acid lengths of its coding protein are between 129 (*TabZIP57*) and 652 (*TabZIP22*), and the average size of amino acid sequences was 314 aa. Isoelectric points of those proteins ranged from 4.36 (*TabZIP208*) to 12.14 (*TabZIP140*). The MW range from 14,098.74 Da (*TabZIP57*) to 68,897.15 Da (*TabZIP22*), with an average value of 33,878.42. The subcellular location shows that all *TabZIP* genes are locate in the nucleus, which is related to the biological function of the gene family as transcription factors. We found that all of the TabZIP protein’s GRAVY is negative, indicating that these proteins are hydrophilic. Also, these are essential physical and chemical properties as transcription factors.

### 2.2. Phylogeny Analysis of TabZIP

**Figure 1:**
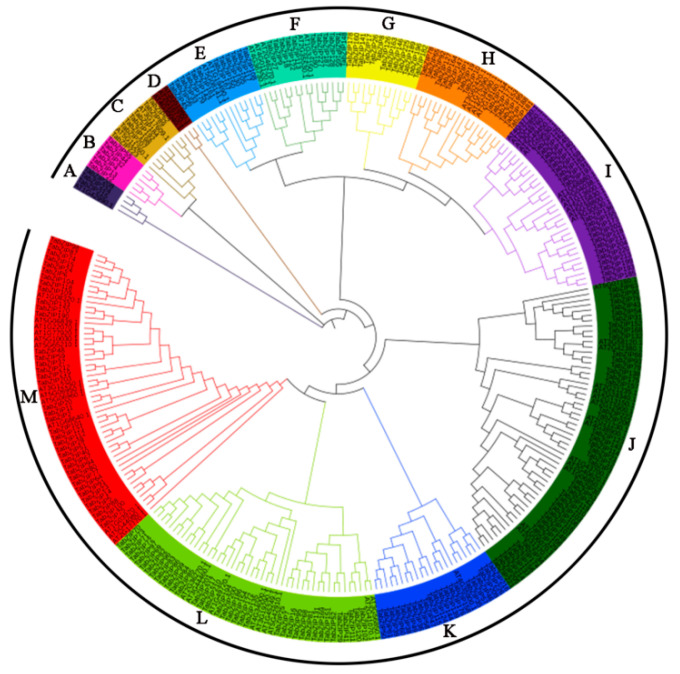
The phylogenetic tree of *bZIP* genes in wheat and *A. thaliana*. Different colors indicate different subfamilies (A–M).

In order to investigate the phylogenetic relationship of TabZIP proteins, the phylogenetic tree was constructed based on multiple sequence alignment of 227 wheat *TabZIP* members and their homologous proteins in *Arabidopsis* (Figure 1). Finally, the *bZIP* genes of wheat and *Arabidopsis* were divided into 13 groups according to the *Arabidopsis* classification system. The group J was the branch with the most members, containing 56 *TabZIP* genes. Intriguingly, it was found that only the bZIP protein of *Arabidopsis* was found in group D. The B and G subfamilies contain only wheat genes and no *Arabidopsis* genes.

### 2.3. Gene Structure and Motif Analysis of TabZIP

**Figure 2:**
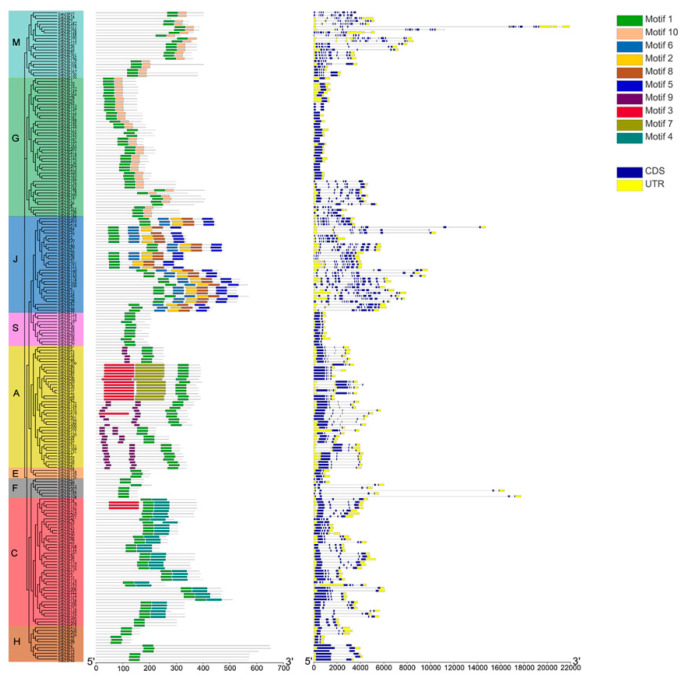
Distribution of introns-Exons and Conserved motifs of the *TabZIP* gene family in wheat. (**Left**) phylogenetic relationship and location of Conserved motifs on each gene and (**right**) distribution of exons/introns of the gene family in wheat.

In order to better understand the functions and phylogenetic relationship of members of the *TabZIP* gene family, structural analysis was performed by comparing each *bZIP* gene in wheat to understand the phenomena of structural diversity. The results showed that 13.6% of *TabZIP* genes (31/227) had no introns in the structure, and these genes that did not contain introns were closely related to each other. The remaining *TabZIP* genes contained 1~14 introns (Figure 2).

In this study, a total of 10 high confidence motifs was predicted, and only one gene (*TabZIP9*) was not identified to contain any motifs. *TabZIP* genes were divided into different subfamilies (A, C, E, F, G, H, J, L, and M) according to the phylogenetic tree and divergence of motifs. Motif composition and distribution of *TabZIP* family members were highly regular, with the number of motifs in all *TabZIP* genes ranging from 1 to 5 (Figure 2). The motif characteristics from the same group were relatively similar, such as S subfamily. However, *TabZIP34*, *TabZIP174*, and *TabZIP137* contain motif3 absent in the other genes in the same subgroup. Motif1 was distributed in all members of the *TabZIP* family, consistent with the result that the Motif1 sequence is a bZIP conserved domain sequence. And about 17.6% of *TabZIP* members (40/227) only contain motif1.

### 2.4. Chromosomal Localization and Synteny Analysis of TabZIP Genes

**Figure 3:**
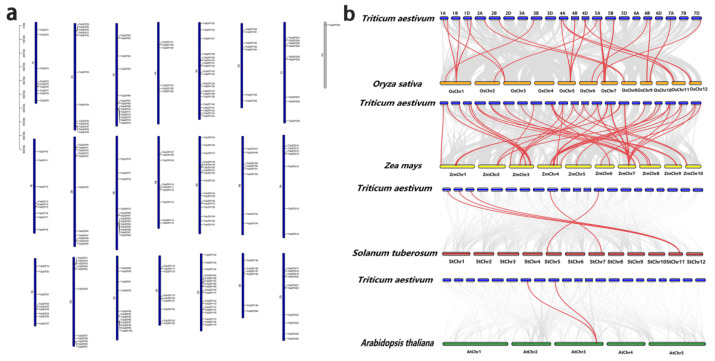
Collinearity analysis of *TabZIP* gene family. (**a**) Chromosome locations of *TabZIP* genes. The chromosome number is on the left side of each chromosome. The scale on the left represents chromosome length. (**b**) Synteny analysis of *TabZIP* genes between *Triticum aestivum* and four representative plant species. Gray lines indicate the collinear bars within the *Triticum aestivum* and other plant genomes, whereas the red lines highlight the syntenic *TabZIP* gene pairs.

All the *TabZIP* genes were located on 21 chromosomes, except for *TabZIP227* (Figure 3a). Each chromosome contains some bZIP genes, but they are unevenly distributed and vary widely. Chromosome 5A contained the largest number of *TabZIP* genes (17.6%), whereas chromosome 1A contained only nine members (3.9%). Chromosomes 5A, 5B, and 5D contain denser genes than others do in the same genome. Moreover, most of the genes were distributed near the ends of the chromosomes.

Besides, to understand the evolutionary relationship of the *bZIP* family in wheat, collinearity relationships were displayed by comparing wheat with four other species. These species include two dicotyledons (*Arabidopsis* and potato) and two monocotyledons (maize and rice). A total of 2, 5, 38, and 22 homologous pairs were identified in wheat and these species, respectively (Figure 3b).

### 2.5. Expansion and Evolutionary Analysis

**Figure 4:**
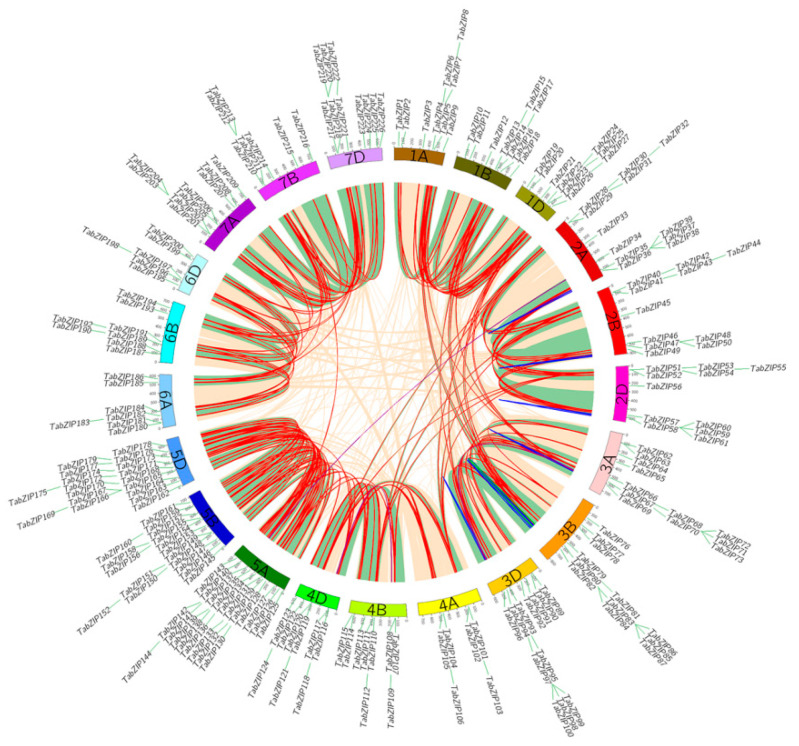
The syntenic pairs of wheat *TabZIP* genes from different duplication mode diagrams. Syntenic pairs from different duplication modes were linked by corresponding color lines. Red, blue, purple, and green represent syntenic regions with segmental, tandem, transposed, and proximal duplication. Others syntenic pairs genes were shown with pale yellow lines.

Gene duplication plays a crucial role in genome evolution. In this study, 227 duplicated genes in *TabZIP* genes were identified, including whole genome duplication (WGD), proximal duplication (PD), tandem duplication (TD), and transposed duplication (TRD). There were 213 duplicated genes (223 pairs) in wheat genome (Figure 4). About 95% of the duplicated genes were identified as WGDs (211 gene pairs), only one PD gene pair (*TabZIP84* and *TabZIP86*) was identified. The remaining duplicated genes were TDs (8 gene pairs) and TRDs (3 gene pairs). The non-synonymous mutation rate to synonymous mutation rate (Ka/Ks) is essential for exploring genomic evolution [33]. The results showed that the Ka/Ks ratios of all wheat *bZIP* genes are from 0 to 1.1387, and only 3 pairs of genes’ ratios were greater than 1 (Table S2). The earliest genetic differentiation appeared 76.7703 million years ago.

### 2.6. Cis-acting Element Analysis

**Figure 5:**
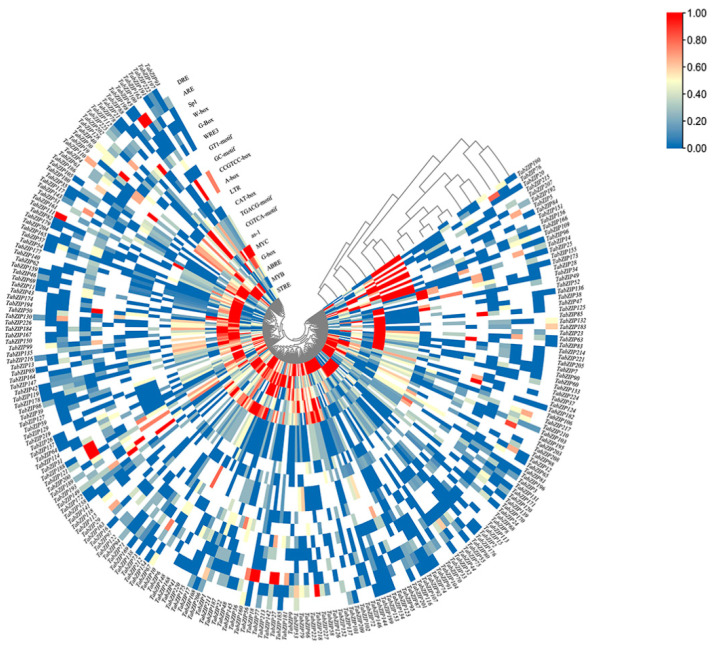
The cis-element of promoter in *TabZIP* gene family. The depth of the color represents the number of times the cis-element occurs.

Promoter cis-element analysis provides convenience for understanding gene function and spatial specific expression. There were many cis-elements in the upstream sequence of wheat *TabZIP* genes (Figure 5). In addition to cis-elements, such as CAT-box and CCGTCC-box, related to growth and development, there were also many photo-responsive elements, such as SP1, GT1-motif, and G-box and hormone-responsive elements, such as TGACG-motif, CGTCA-motif (methyl jasmonate-induced), ABRE (ABA response element), and as-1 (Auxin response element). However, the most common classes of cis-elements were those that respond to abiotic stresses, such as low-temperature response (LTR), involved in anaerobic inducible enhancer (GC-Motif), antioxidant response (ARE), dehydration stress (DRE), drought (MYB, and MYC), and osmotic stress response. Finally, it was not difficult to find that the high-frequency cis-acting elements in the promoter of *TabZIP* genes concentrated in stress-related elements, such as drought and osmotic stress.

### 2.7. Expression Analysis of TabZIP Gene and RNA-Seq under Abiotic Stress

**Figure 6:**
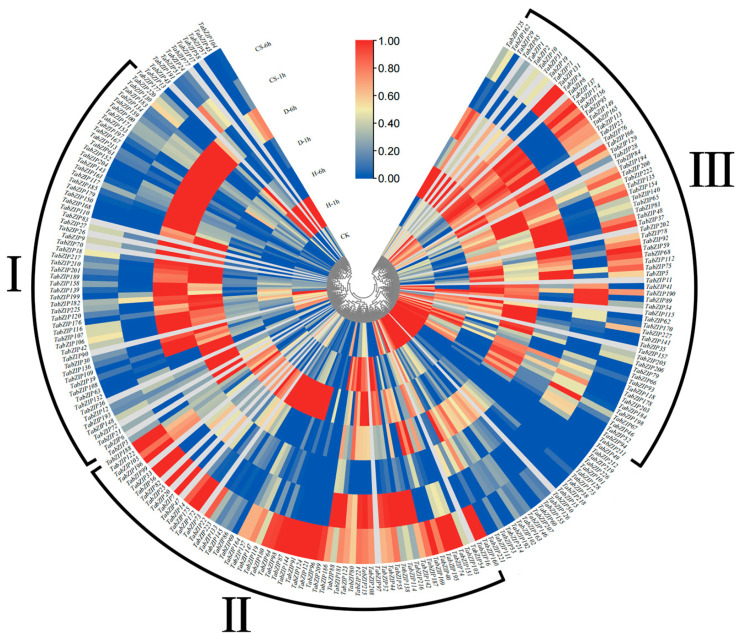
Transcriptional profiles of TabZIP gene family members in wheat under heat stress (H-1/H-6), drought stress (D-1/D-6), and combined stress (CS-1/CS-6). Grids with colors indicate low/down expression (blue), high/up expression(red), and non-expression/no change (yellow). According to the similarity of expression characteristics, some genes were classified into Group I, Group II and Group III respectively.

The potential role of wheat bZIP family in different abiotic stresses was analyzed based on RNA-Seq data. The RNA-Seq data were analyzed under the drought and high temperature of about 40 °C. Transcriptional levels of TabZIP genes at 1 h and 6 h after treatment were revealed. These treatments included drought for 1 h (D-1) and 6 h (D-6), high temperature for 1 h (H-1) and 6 h (H-6) at 40 °C, and the combination of the two stresses for 1 h (CS-1) and 6 h (CS-6). The TabZIP genes had a high expression in each type or time for treatment (Figure 6). Among them, red represented high/up-regulated expression of the genes, low/down-regulated expression genes were shown in blue, and yellow represents no significant difference in gene expression. The genes in group Ⅰ were significantly up-regulated under drought treatment. Except for Ⅲ groups of genes, the genes with high expression under heat stress were lower in drought condition. The number of differentially expressed genes was similar to heat treatment. It was noteworthy that TabZIP179 was the most significantly up-regulated gene under drought treatment, and it was also significantly up-regulated in H-6, while the most significantly up-regulated gene in H-1 was TabZIP191. In CS-1, almost all genes had the low expression, and genes with high expression in CS-6 were mainly in group Ⅱ. In group Ⅲ, the highly expressed genes were densely distributed in all treatments except CS-1.

**Figure 7:**
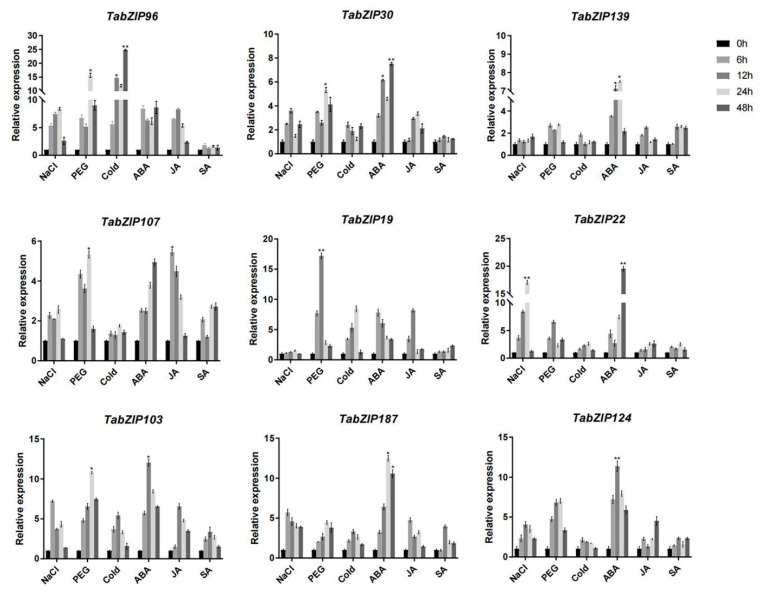
Relative expression analysis of 9 genes in the TabZIP gene family under cold stress, drought stress, salt stress, ABA treatment, JA treatment, and SA treatment in wheat. All values in the figure are mean ± SD (*n* = 3), p values (versus 0 h) were corrected with ANOVA, *p* < 0.05 indicates significant difference, indicated by *; *p* < 0.001 indicates high significance, indicated by **.

In general, gene expression level and pattern indicate gene function. To clarify the expression characteristic of wheat TabZIP gene family. We examined the relative expression levels of genes in each subgroup of the TabZIP under abiotic stresses (salt, drought, and cold) and hormones (ABA, JA, and SA), as shown in Figure 7. The expression levels of *TabZIP96* and *TabZIP22* were up-regulated under salt stress, and their expression trends were similar, which increased first and then decreased, and reached the peak at 24 h. Most genes respond to drought. The relative expression of TabZIP19 and TabZIP103 after PEG treatment was significantly increased by 10 and 15 times, respectively. The expression of the Only 3 genes (*TabZIP96*, *TabZIP19*, and *TabZIP103*) were significantly induced by low temperature. Besides, the hormone treatment results showed that ABA-induced all genes, and some responded rapidly to up-regulation in the early stage, and some responded violently in the late stage. Most genes were also involved in the response of JA, such as *TabZIP96*, *TabZIP107*, and *TabZIP19*. While the expression level changes of most genes after SA treatment were not significant.

### 2.8. Overexpression of TaABI5 in A. thaliana Strengthens Plant Freezing Tolerance

**Figure 8:**
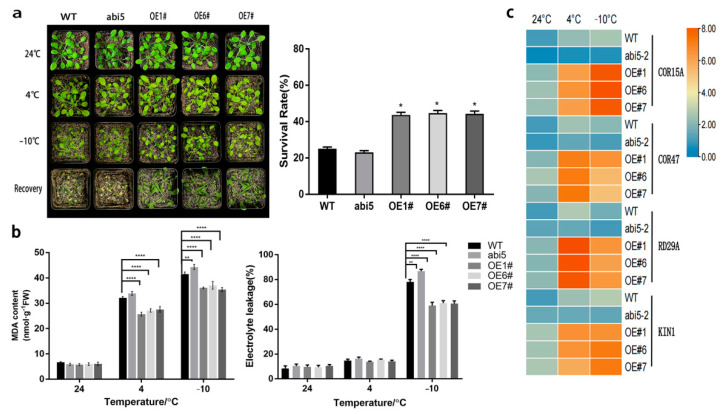
Analysis of cold resistance and expression of key stress response genes in transgenic plants. (**a**) Phenotype and survival rate of *Arabidopsis* lines under freezing stress. (**b**) MDA content and electrolyte leakage were measured. (**c**) Heat maps of stress response genes expression at 24 °C, 4 °C and −10 °C in different plant lines, respectively. All values in the figure are mean ± SD (*n* = 3), *p* values (versus WT) were corrected with ANOVA, * *p* < 0.05, ** *p* < 0.01, and **** *p* < 0.0001.

Due to the particular function of genes in subgroup A under abiotic stress, we cloned the entire length of gene TaABI5 (TabZIP96) in group A. In order to investigate the potential effect of TaABI5 on low-temperature tolerance in plants, we constructed overexpression vector 35S: TaABI5 and transformed it into wild-type A. thaliana plants (WT; col-0). Overexpression of the TaABI5 gene in A. thaliana plants resulted in significantly enhanced freezing stress resistance (Figure 8). There was no significant phenotype difference in all lines at 4 °C, but leaf curl and wilting occurred in all lines when the temperature dropped to −10 °C. It was easier to be observed that the mutant abi5 with functional loss showed a cold-sensitive phenotype at −10°C and that the OE lines were slightly stronger than the wild-type. After seven days of recovery growth, survival rates of the three transgenic lines were 43%, 45%, and 44%, respectively, significantly higher than that of WT plants (25%), as shown in Figure 8a. The malondialdehyde (MDA) contents of the three transgenic lines were 33, 31, and 33 nmol g^−1^ FW after treatment at −10°C (Figure 8b), respectively, which were lower than that of the wild-type lines (42 nmol g^−1^ FW). Electrolyte leakage rates, another indicator of membrane permeability, were 60%, 62%, and 61% in OE lines, which were lower than WT lines (78%), as shown in Figure 8b.

Transcriptional levels of low temperature response-related genes were detected by qRT-PCR. As shown in the Figure 8, transgenic plants overexpressing TaABI5 may up-regulate the expression of several key stress-responsive genes compared with the wild type under normal conditions. These stress-responsive genes include RD29A, COR47, COR15A, and KIN1 (Figure 8c). As expected, the expression of these genes was down-regulated in abi5 mutant plants. After 4 °C treatment for three days, these stress-responsive genes were significantly induced. Thereinto, AtRD29A gene was highly up-regulation. AtCOR15A was highly expressed under −10 °C stress. Furthermore, the expression of stress-responsive genes in transgenic plants was more than those of wild-type.

### 2.9. TaABI5 Regulates Low-Temperature Stress Response by Participating in ROS Metabolism

**Figure 9:**
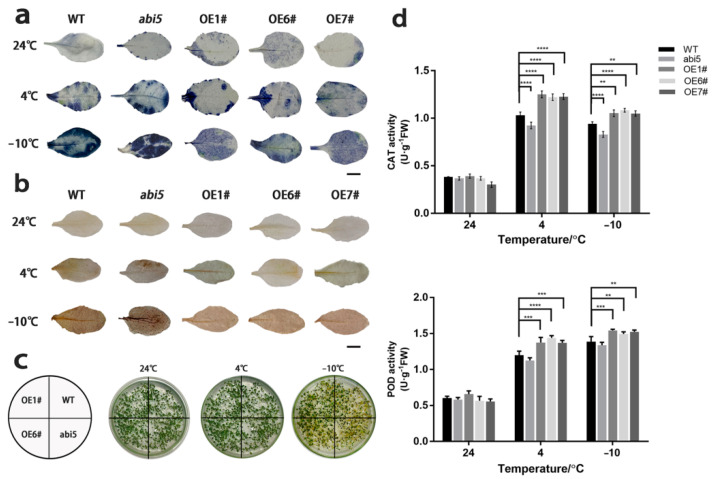
Overexpression of TaABI5 in *A. thaliana* enhanced tolerance to freezing stresses and ROS-scavenging ability. (**a**) Histochemical staining assays with NBT. (**b**) Histochemical staining assays with DAB. (**c**) The 2-week-old transgenic, wild type, and abi5 seedlings were exposed to freezing treatment. The plants were acclimated at 4 °C for 3 d, then treated at −10 °C for 2 h, and restored to normal culture for 7 d. (**d**) CAT and POD of *Arabidopsis* lines under freezing stress. All values in the figure are mean ± SD (*n* = 3), *p* values (versus WT) were corrected with ANOVA, ** *p* < 0.01, *** *p* < 0.001, and **** *p* < 0.0001.

In order to study the effect of TaABI5 on plants under low temperature stress, four weeks old A. thaliana were subjected to low temperature stress. Cell membrane damage caused by excessive ROS is a fundamental cause of osmotic stress, such as cold stress [45]. We examined the levels of superoxide (O^2−^), hydrogen peroxide (H_2_O_2_), and antioxidant enzymes activities of abi5, transgenic plants and wild-type *Arabidopsis* under cold stress by 3,3′-diaminobenzidine staining (DAB) and Nitroblue tetrazolium staining (NBT). CAT and POD showed high activity in OE lines, which was consistent with DAB and NBT staining results. As shown in Figure 9, the staining of NBT and DAB in transgenic lines was weaker than that of WT and abi5.

### 2.10. Subcellular Localization of TaABI5 and Y2H Assay

**Figure 10:**
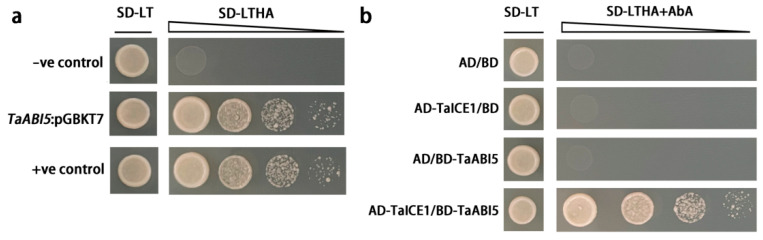
Y2H assay of interaction between TaICE1 and TaABI5. (**a**) Transcription activation assay of TaABI5 in yeast. (**b**) TaICE1 and TaABI5 were co-transformed into yeast. Colonies were assayed for SD/-Leu-Trp autotrophy and SD/-Leu-Trp-His-Ade with 40 mM Aureobasidin (AbA) autotrophy.

The bZIP genes perform the molecular function as a transcription factor. It can activate the transcription of downstream genes. The transcription activation of TaABI5 has been detected. Full-length TaABI5 fused with GAL4 DNA-binding domain (BD-TaABI5) was transformed into yeast strains. As shown in Figure 10a, no yeast colony growth was observed in the negative control (BD-PGBKT7 empty vector) on the SD/-Trp/-His/-Ade medium, but BD-TaABI5 activated the expression of reporter genes with strong transcription activation activity.

In order to explore the regulatory mechanism of TaABI5 in plant response to cold stress, the full-length TaICE1 into the Gal4 activation domain of the prey vector (AD-TaICE1). BD-TaABI5 and AD-TaICE1 were co-transformed into yeast strain AH109. As shown in Figure 10b, only the yeast co-transferred by TaABI5 and TaICE1 could survive on QDO medium with 40 mM Aureobasidin A (AbA). The results demonstrate that TaABI5 physically associated with TaICE1.

### 2.11. Analysis of the Subcellular Localization of TaABI5

**Figure 11:**
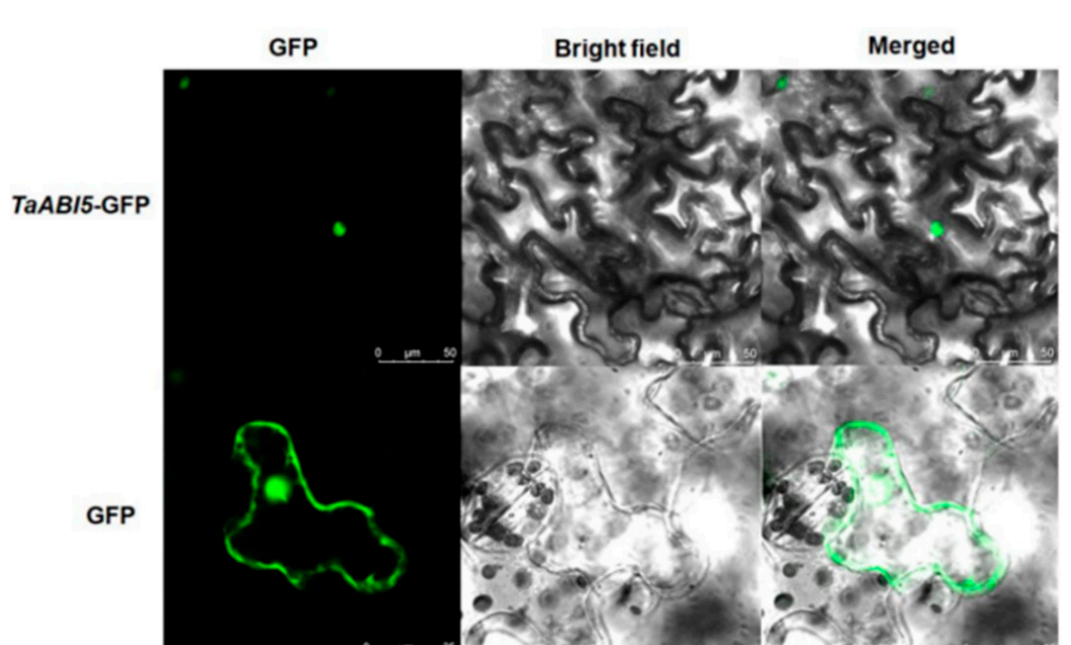
Fluorescence of TaABI5 in tobacco leaf cells. Subcellular localization of TaABI5 (**top**) and empty vector (**bottom**) in dark field, bright field, and merged field. Scale bars = 25 µm.

The subcellular localization of TaABI5 was explored to understand more functions. TaABI5: GFP was transformed into the leaf cells of *N. benthamiana*. As shown in Figure 11, only the nucleus of tobacco cells emitted green fluorescence in the confocal laser imaging, which indicated that TaABI5 fused with green fluorescence protein was located in the nucleus.

## 3. Discussion

As wheat is a very important economic crop, it is necessary to study the regulation mechanism of its frost resistance. The *bZIP* genes is a large transcription factor in plants and can participate in a variety of physiological and biochemical reactions [46]. The family also plays an important role in regulating plant stress tolerance [47]. This study focused on the identification and analysis of *bZIP* gene family members in wheat, and attempted to explore the effect of *bZIP* gene on cold tolerance of wheat.

In the previous report by Agarwal et al., 191 *TabZIP* genes were identified [48]. Nevertheless, 36 new genes have been added to the current version (Table S1). In this study, 227 *TabZIP* gene family members were identified and analyzed from the wheat genome database. The results were compared to those of previously identified species *Arabidopsis thaliana* (75 genes) [3], *Solanum lycopersicum* (69 genes) [49], *B.distachyon* (96 genes) [50], *Oryza sativa* (89 genes) [9], *Zea mays* (125 genes) [51], *Glycine max* (131 genes) [52], *Coniothyrium minitans* (34 genes) [53], *Vitis vinifera* (55 genes) [54], *Malus domestica* (116 genes) [55], *Fragaria vesca* (50 genes) [20], *Sorghum bicolor* (92 genes) [47], and *Prunus persica* (47 genes) [56]. The number of *bZIP* gene family members increased, but was relatively small compared with *B. napus* contain 247 *bZIP* genes [57]. It is not unusual for gene families to change in size due to gene duplication, deletion, pseudogenization, and functional diversification [58]. The *bZIP* gene of rice [9], apple [55], and grapevine [54] can be divided into the same 10 subgroups as that of *Arabidopsis* [46], which is basically consistent with the classification of wheat in our study. According to its evolutionary branch and gene motifs, this study divided wheat *bZIP* gene into nine groups for subsequent specific analysis (Figure 1). Different branches indicate functional differences or that these subfamilies have specific functions [59]. Group D contained only *AtbZIP* genes but no *TabZIP* genes, indicating that the *TabZIP* genes in these groups might have been lost or differentiated into other groups during the evolution of wheat [60]. Groups B and G contain only the TabZIP gene, suggesting that this clade may be specific to wheat [61].

Gene duplication can provide the evolutionary potential for new functions in species [62]. This study revealed the gene duplication patterns of the *bZIP* gene family in wheat and visualized their chromosomal locations, as shown in Figure 3. The *TabZIP* was a large gene family distributed on each chromosome (Figure 3a). The *TabZIP* genes in wheat is closely related to monocotyledonous crops, such as potato and rice. Most plants have experienced a genome wide duplication event or a segmental duplication event [63], a large-scale chromosome doubling event that results in the retention of a large number of chromosomes doubling segments in the genome [64]. According to predecessors, it is speculated that hexaploid wheat is a cross between tetraploid wheat and Aegilops [65]. Therefore, hexaploid wheat has a large number of the oldest and most conserved fragment replication events [66]. Segmental genome duplications or whole genome duplication events may explain the expansion of the *bZIP* gene family [57]. This study identified 211 gene pairs with WGD, accounting for about 95% of the total gene pairs. Wheat also contained eight gene pairs with TD (Figure 4). The eight gene pairs (14 genes) formed a gene cluster, in which genes had similar sequences and similar functions [67]. Notably, only one pair of genes had PD events. This means that these two genes (*TabZIP84* and *TabZIP86*) may have greater adaptive ability under stronger selection pressure [68]. Ka/Ks ratio can determine whether there is selection pressure on the protein-coding gene, and the ratio is essential for exploring genomic evolution [69]. Most genes were purifying selection (Ka/Ks < 1) and a few were positively selected (Ka/Ks > 1) [69]. The study results indicate that 98.6% of gene pairs had a Ka/Ks ratio less than 1, indicating the evolution of the wheat *TabZIP* family was relatively conserved [70]. This result also indicated that there was no significant functional division after fragment duplication and tandem duplication, suggesting that purifying selection played an important role in the evolution of the wheat genome. However, there were five genes (three gene pairs) with Ka/Ks ratios greater than 1, which were supported by Darwinian selection and led to adaptive evolution [71]. And these genes were strongly positively selected, which have recently evolved rapidly and were of great importance to the evolution of species [72]. Overall, the *TabZIP* gene family members in wheat genomes were relatively evolutionarily conserved. This provided a theoretical basis for further exploring the function and properties of *TabZIP* genes.

In order to explore the specific expression pattern of *TabZIP* gene in wheat, transcriptome analysis found that the expression of the entire gene family responds to abiotic stresses such as drought and heat to varying degrees (Figure 6). The expression levels of most *TabZIP* genes were significantly up-regulated after drought and heat combined stress. At the same time, their promoter cis-acting elements were analyzed and found to contain a variety of components in response to abiotic stress, including low temperature (Figure 5). These results suggest that *TabZIP* gene has a high probability of responding to environmental stress and may be involved in the regulation of plant response to abiotic stress [73]. In order to confirm the reliability of the above analysis and verify the above speculation, we applied three kinds of abiotic stress and three kinds of hormone treatment to indoor wheat seedlings at the three-leaf stage [74]. The qRT-PCR data and promoter cis-acting elements analysis showed that the *TabZIP* genes were up-regulated after ABA treatment. We also found that cis-acting elements associated with ABA response were highly enriched in promoters of a large number of genes (Figure 5). This result is consistent with the biological identity of *bZIP* as a transcription factor [75]. The *bZIP* genes are involved in ABA signaling and ABA plays an important role in the process of plant resistance to stress [76]. Therefore, we speculate that the bZIP gene also has a prominent contribution in regulating plant stress resistance. Cis-acting element results also found many elements related to abiotic stress response, such as LTR, ARE, DRE, MYC, and MYB. The results showed that there were a large number of photoinduction-related elements in the *TabZIP* gene promoter, which may be closely related to light response and light sensitivity. The functions of light response need to be further explored.

Subsequently, we examined the expression levels of *TabZIP* genes (one gene from each subfamily was randomly selected) under different abiotic stress treatments. *TabZIP* gene family showed different expression patterns in response to salt, drought, and low temperature (Figure 7). The expression of *TabZIP107* and *TabZIP30* increased significantly after 48 h of stress treatment, suggesting that these two genes may play a role in the late stage of salt stress. In drought treatment, all genes were up-regulated in different amplitude, and the gene (*TabZIP19*) in group G was up-regulated 17 times rapidly after drought stress. The above findings deserve further confirmation and exploration. Most genes were insensitive to cold stress. However, the gene *TabZIP96* with differential expression appeared in group A, and its expression was significantly up-regulated by more than 20 times of low temperature stress. We predict that this gene may play a role in the later stage of plants responding to low temperature stress, providing a good adaptive basis for plants to tolerate freezing stress conditions at lower temperatures [77]. Simultaneously, to further explore the relationship between *TabZIP* genes and hormone regulation, the expression pattern of *TabZIP* gene family was analyzed under different hormone treatments (Figure 7). All genes were induced under ABA, and the expression trend was similar to that under JA treatment. These results suggest that *TabZIP* genes may play a pivotal role in these two hormone pathways, and JA responsive genes are also regulated by bZIP transcription factors [78]. This result is consistent with the existence of a large number of JA related cis-acting elements, such as TGACG-motif and CGTCA-motif in Figure 5. ABI5 encodes a bZIP transcription factor that physically interacts with JAZ1 in the JA pathway [79]. This confirmed that *bZIP* gene is involved in the regulation of crosstalk of two hormone signals [80,81].

In order to deeply explore the effect of *bZIP* gene on cold resistance of wheat, we screened a gene involved in low temperature response through real-time fluorescence quantitative results and verified the biological function of the gene (Figure 7). *TabZIP96* (*TaABI5*), a group representative member of the *TabZIP* gene family, was cloned as a homologous gene of *AtABI5* in wheat [82]. The gene response to various stress conditions and showed obvious transcriptional characteristics. Phenotypic analysis and related physiological indicators of cold resistance showed that overexpression of *TaABI5* could enhance cold resistance of transgenic *Arabidopsis thaliana* (Figure 8).

The cold tolerance of plants was negatively correlated with the degree of cell damage. The stronger the plant’s tolerance, the lower the degree of cell damage. The root cause of cell destruction is the accumulation of ROS [83,84]. High levels of O^2−^ and H_2_O_2_ can lead to accumulation of ROS [85]. DAB and NBT staining can indicate O^2−^ and H_2_O_2_ levels [86]. And the higher the level of cell damage, the deeper DAB and NBT staining [87]. Both CAT and POD activities were stronger than those of wild-type and mutant plants, indicating a strong ROS scavenging ability [88] (Figure 9d). Staining depth of each line of transgenic plants were lower than that of the WT, and the mutants with functional loss were the darkest in Figure 9. Which means that plants have a strong ability to scavenge reactive oxygen species to resist oxidation [89]. The low temperature destroys the structure of the cell membrane [90,91]. Freezing damage to plant cells is more serious, so that intracellular regionalization is highly damaged [92]. Electron leakage rate can reflect the disorder degree of ion transfer reaction of cells, and the side exposure of membrane permeability [87,93]. Otherwise, it was demonstrated that overexpression of *TaABI5* enhanced the expression of downstream cold-responsive genes in *Arabidopsis thaliana*, especially under low temperature and freezing conditions (Figure 8c). These results once again proved that functional *TaABI5* could enhance the frost resistance of plants.

By observing the phenotype of each line under freezing stress, the growth state and survival rate of OE line were better than other lines (Figure 9c). To further explore how *TaABI5* is involved in the cold resistance pathway, we found that TaABI5 physically interacts with TaICE1 in the yeast two-hybrid system (Figure 10b). Subcellular localization experiments confirmed that *TaABI5* was located in the nucleus, which was in line with the biological role of transcription factor (Figure 11). This suggests that these two proteins recognize each other in the nucleus and bind to form protein complexes that are involved in each other’s regulatory pathways, although their respective functions are different. We speculated that TaABI5 and TaICE1 jointly regulate the tolerance of wheat to low-temperature stress. Based on these results, we concluded that the effect of *TaABI5* on plant cold stress tolerance may be realized through the interaction with *TaICE1*. Analogously, research has shown that MdABI4 interacts with MdICE1 protein modules jointly regulating growth of plants [94]. ABI5 in *Arabidopsis* demonstrates physical interaction with MED25 [95]. MED25 is involved in different regulatory pathways as an intermediary subunit, its functions are complex [96]. Therefore, the functions of ABI5 in other aspects and its interaction network need to be further explored and studied. Studies have shown that ABI5 binds directly to the promoter regions of *EM1* and *EM6*, promoting their transcription [97]. EM1 and EM6 are late embryogenesis abundant proteins (LEA), which belong to a class of cold-responsive protein [93]. Furthermore, ABI5 can regulate the expression of *RAB18*, *COR15A* and *COR6.6* [98]. *RAB18* has a certain sense in the low temperature resistance pathway [99]. *COR15A* and *COR6.6* are well-known cold-response genes [100]. The cold resistance of *TaABI5* may also directly affect the expression of these cold tolerance genes. The cold-resistant regulatory network of TaABI5 in wheat needs to be further confirmed and supplemented. In conclusion., it can be indicated that *ABI5* had an important position in plant under cold stress, and *TaABI5* also plays a certain function in cold response pathway of wheat.

## 4. Materials and Methods

### 4.1. Identification of the Members of TabZIP Gene Family in Wheat

The Hidden Markov model of *bZIP* domain (PF00170) was downloaded by PFAM (protein domain family) database (http://pfam.xfam.org, accessed on 11 October 2021), and the *bZIP* genome sequence information of wheat genome 2.0 was downloaded by IWGSC (http://www.wheatgenome.org/, accessed on 11 October 2021). To identify *TabZIP* genes in wheat, Hmmsearch tool was used to retrieve a domain similar to the bZIP domain in wheat, and the value was set to 1×10^−5^. These protein sequences were submitted to Pfam and CDD (Conserved Domains Database, https://www.ncbi.nlm.nih.gov/Structure/cdd/cdd.shtmlis, accessed on 11 October 2021) to remove redundant sequences for further verification of the inclusion of the *bZIP* domain.

### 4.2. Sequence Analysis of TabZIP Genes in Wheat

All CDS sequences of *TabZIP* genes were submitted to ExPASy (https://www.expasy.org, accessed on 11 October 2021) for gene length, amino acid length, relative molecular weight, isoelectric point, hydrophilicity, stability, and other physicochemical properties analysis. The gff3 file was used to extract the intron-exon and UTR (non-coding region) distribution and other genes structure information, which was finally visualized by TBtools [101]. And the software MEME was used to analyze the conservative motifs of the proteins [102]. The maximum number of motifs to be found was 10, motif width was 6–20, and other parameters were default values. Promoters affect gene function to a certain extent. The 2000 bp sequence upstream of the *TabZIP* genes transcription start site was submitted to PlantCARE (http://bioinformatics.psb.ugent.be/webtools/plantcare/html, accessed on 11 October 2021) to analyze the promoters enrichment type of the gene family members.

### 4.3. Phylogenetic Analysis and Chromosomal Location

MUSCLE in MEGA Version 7 software was used to align the full-length sequence of *TabZIP* gene family members, and 1000 was used as the bootstrapping to build the neighbor joining tree [103]. The locations and chromosome lengths of all *TabZIP* genes were obtained from the Ensembl Plants database and the data were located using TBtools. In addition, to study the evolutionary relationship of *bZIP* genes between wheat and *Arabidopsis*, the *bZIP* gene sequences of *Arabidopsis* and wheat were used to map evolutionary trees. The genome and protein sequences of them were downloaded from the Ensembl Plants database using iTOL (https://itol.embl.de, accessed on 12 October 2021) for beautification.

### 4.4. Duplication, Ka/Ks, and Synteny Analysis

Blastall tool was used to blast the CDS sequence of 227 genes of *TabZIP*, and the expectation value is 1 × 10^−20^ [104]. MCScanX was used to analyze gene replication and synteny events. The proteins sequence of Arabidopsis (*A. thaliana*), potato (*S. tuberosum*), maize (*Z. mays*), and rice (*O. sativa*) were downloaded from Ensembl Plants. The evolutionary relationship between genes is performed by synonymous substitution rate (Ks) and nonsynonymous substitution rate (Ka). Ka/Ks ratio in wheat was calculated by KaKs_calculator [105]. All duplicated gene pairs of *TabZIP* genes in wheat were classified into the following four categories: segmental, tandem, proximal, and dispersed duplications. Finally, collinear regions and names of *TabZIP* gene family were visualized by Circos software. The divergence time of gene duplication (MYA) was calculated by the formula T = Ks/2λ with a value of =6.5 × 10^−9^ synonymous substitutions per site per year [106,107].

### 4.5. Expression Analysis Based on RNA-Seq

NCBI (National Center for Biotechnology Information) database was used to obtain the transcriptome data (accession number: SRP045409), and transcriptome enrichment analysis was performed on members of the TabZIP family. Control (SRR1542405, SRR1542404), drought treatment for 1 h (SRR1542407, SRR1542406), 6 h (SRR1542409, SRR1542408), heat treatment for 1 h (SRR1542411, SRR1542410), 6 h (SRR1542411, SRR1542410), drought and heat combined treatment for 1 h (SRR1542415, SRR1542414), and 6 h (SRR1542417, SRR1542416). HISAT software was used to map the reads from all samples to the wheat genome [108].

### 4.6. Plant material and Treatments

Winter wheat (*Triticum aestivum*) cultivar Dongnongdongmai1 (Dn1) was obtained from the Wheat Breeding Institute of Northeast Agricultural University, Harbin, China. 100 uM ABA (Abscisic-acid), 100 uM MeJA (Methyl Jasmonate), 100 uM SA (Salicylic acid) [109,110], and Cold (4 °C), 200 mM NaCl solution and 20% PEG solution were used to treat wheat seedlings at the tri-leaf stage for 48 h [111]. The seedlings grew in a greenhouse with 22 h of light and 16 h of dark. Total RNA was extracted from the leaves of unstressed and stressed wheat seedlings at 0, 6, 12, 24, and 48 h.

*Arabidopsis* was selected as the Columbian type. The abi5 (SALK_013163, Col-0 background) mutant was a kind gift from Dr. Gang Wu (State Key Laboratory of Subtropical Silviculture, School of Agriculture and Food Sciences, Zhejiang Agriculture and Forestry University) [112]. The culturing condition was 24 C, 16/8 photoperiod [113]. Four-week-old *Arabidopsis thaliana* was treated at 4 °C for 3 days, and then the plants were subjected to −10 °C for 2 h. Finally, phenotypic analysis was performed 7 days after resuming culture conditions [44].

### 4.7. Total RNA Extraction and qRT-PCR

Total RNA was extracted with Trizol [114], reverse transcribed to cDNA with the TransScript One-Step gDNA Removal and cDNA Synthesis SuperMix Kit (Applied Biosystems, Shanghai, China). One gene from each subfamily was selected for qRT-PCR under different treatments to analyze the expression characteristics of *TabZIP* gene family members. As shown in Table S3, the primers were designed on NCBI. The ChamQ™ Universal SYBR qPCR Master MIX (Vazyme, Nanjing, China) was used. Three biological replicates were set for each sample. It was used to analyze the expression characteristics of *TabZIP* genes under different stresses. The relative expression level was calculated using the 2^−∆∆ct^ method.

### 4.8. Cloning of TaABI5 and Plant Transformation

The full-length CDS sequence of *TaABI5* was cloned and inserted into a plant expression vector (Pcambia230035Su) by user method so that the expression of *TaABI5* was driven by CaMV35S promoter, and the recombinant plasmid of plant overexpression vector was transformed into Agrobacterium tumefaciens strain EHA105. The 35S:*TaABI5* constructs was transformed into *Arabidopsis* wild-type (Col-0) plants. Finally, 1/2MS medium containing kanamycin resistance was used to screen T3 generation positive plants for subsequent experiments.

### 4.9. Determination of Physiological Indices and ROS Metabolism Assays

Malondialdehyde (MDA) content in *Arabidopsis* leaves was detected using the method previously reported [115]. Detection of catalase (CAT) activity according to the method of Aebi [116], Peroxidase (POD) activity was measured by the method of Chance [117]. In addition, plant resistance to low temperature stress was related to the accumulation of reactive oxygen species (ROS) [1]. 3,3′-diaminobenzidine (DAB) and nitro blue tetrazolium (NBT) tissue staining can understand ROS accumulation by visualizing H_2_O_2_ and O^2−^ content, specific methods and steps have been described by predecessors [118].

### 4.10. Statistical Analysis

Graphpad Prizm 7.0 statistical software was used for data analysis, ANOVA was used to test significance, * *p* < 0.05, ** *p* < 0.01, *** *p* < 0.001, and **** *p* < 0.0001. All experiments were repeated three times. The values presented the means ± one standard deviation (SD) of three biological replicates.

### 4.11. Two-Hybrid Assay and Subcellular Localization

The CDS sequences of *TaABI5* and *TaICE1* were cloned and full-length cDNA were respectively inserted into bait vector pGBDT7 and prey vectors pGADT7 for yeast two-hybrid experiments. As both of them are transcription factors with transcriptional activation activities, transcriptional activation experiments are indispensable. The recombinant pGBDT7:*TaABI5* was transformed into strain AH109 (Takara, Shiga, Japan).

In the yeast two-hybrid experiment, recombinant plasmids pGADT7:*TaICE1* and pGBDT7:*TaABI5* were co-transformed into yeast strain AH109 and cultured on SD/-Trp-Leu medium for 60–80 h, then transferred to SD/-Trp-His-Leu-Ade medium with AbA (400 ng/mL) (Takara, Shiga, Japan) for 80–90 h.

The full-length *TaABI5* sequence was inserted into the green fluorescent protein (GFP) vector. The p*TaABI5*:GFP vector was constructed using the above methods. Nicotiana benthamiana epidermal cells were infected by Agrobacterium-mediated. The transient expression of TaABI5 was detected using laser confocal microscopy.

## 5. Conclusions

A total of 227 *TabZIP* genes were identified in wheat, adding 36 new genes from previous studies. The *TabZIP* gene family was divided into nine branches according to the gene structure and motif. The promoters of these genes contained many cis-acting elements related to abiotic stress and showed different expression patterns under different abiotic stress conditions. We demonstrated that *TaABI5* increased the expression of downstream stress-response genes, and that overexpression of *TaABI5* enhanced the resistance to low temperature and freezing stress in transgenic plants. For another, TaABI5 located in the nucleus and physically interacted with TaICE1. These results indicated that *TabZIPs* had certain significance in wheat under low temperature stress, and laid a good foundation for further exploring the regulation mechanism of wheat stress resistance.

## Supplementary Materials

The following supporting information can be downloaded at: https://www.mdpi.com/article/10.3390/ijms23042351/s1.
